# Employability of Persons With Mental Disability: Understanding Lived Experiences in Kenya

**DOI:** 10.3389/fpsyt.2019.00539

**Published:** 2019-07-30

**Authors:** Ikenna D. Ebuenyi, Mònica Guxens, Elizabeth Ombati, Joske F. G. Bunders-Aelen, Barbara J. Regeer

**Affiliations:** ^1^Athena Institute, Amsterdam Public Health research institute, Vrije Universiteit Amsterdam, Amsterdam, Netherlands; ^2^ISGlobal, Hospital Clínic—Universitat de Barcelona, Barcelona, Spain; ^3^Department of Experimental and Health Sciences, Pompeu Fabra University, Barcelona, Spain; ^4^Spanish Consortium for Research on Epidemiology and Public Health (CIBERESP), Instituto de Salud Carlos III, Madrid, Spain; ^5^Department of Child and Adolescent Psychiatry/Psychology, Erasmus University Medical Centre–Sophia Children’s Hospital, Rotterdam, Netherlands; ^6^Users and Survivors of Psychiatry, Nairobi, Kenya

**Keywords:** employability, self-employment, social support, psychosocial disability, Kenya, East Africa

## Abstract

**Introduction:** Globally, mental illness affects social and occupational functioning. We aimed to highlight the barriers to employment experienced by persons with mental disabilities in Kenya and how they manage to find work against all the odds.

**Materials and Methods:** Using a mixed-method study design, we purposely sampled persons with mental illness through networks of persons with psychosocial disabilities (Users and Survivors of Psychiatry and Africa Mental Health Foundation, Kenya). Qualitative data were obtained through in-depth interviews (*n* = 14) and four focus group discussions (*n* = 30), while a researcher-designed questionnaire was used to obtain quantitative data (*n* = 72).

**Results:** We identified five major clusters of barriers to employment: mental illness factors, social exclusion and stigma, work identity crisis, non-accommodative environment, and socioeconomic status. Factors that facilitated employment include self-awareness and acceptance, self-employment, provision of reasonable accommodation, improved health services, addressing discriminatory laws and practices, and social development programs and support. Participants considered psychiatric illness the highest barrier to employment (63.2%), while supportive family/friends were considered the highest facilitator of employment (54.5%).

**Conclusion:** The employment experiences of persons with mental disabilities are influenced by various interrelated factors in their social environment. Proactive social support and affirmative action by government may improve their employment opportunities and quality of life.

## Introduction

Globally, persons with disabilities often experience stigma and social exclusion, which negatively affects major areas of life including access to health care, education, relationships, employment, and social participation (1–6). Studies suggest a two-way relationship between mental illness and poverty and show how they reinforce each other (1, 7).

For the individual with mental illness, this is not only a source of disability (8) but also a limitation to obtaining relevant help. This is because mental illness is often not considered as a disability, and individuals affected by it experienced both overt and covert discrimination in employment (9, 10) than do persons with other disabilities. The discrimination and stigma associated with mental illness often affect the decision to disclose, which makes it impossible to obtain reasonable accommodation in education and employment. The dilemma of disclosure and identifying as an individual with mental or psychosocial disability also limits employment opportunities (11–13). In high-income countries, supported employment practices for vocational rehabilitation of individuals with mental disabilities are associated with improved employment outcomes (14–17). Evidence of the usefulness of supported employment and modified work environment for the benefit of individuals with mental illness has been document in the UK, USA, and Netherlands (14–17). Also, self-employment (where an individual works for self or owns the business) as a useful employment option for persons with psychiatric disabilities has been reported in the USA (18). There are few examples of vocational rehabilitation for persons with mental illness in low-income settings in Africa (19). In low-income settings, proximal challenges of interrupted education and poverty also affect their ability to set up their own business, thus ruling out the self-employment that may have served as an alternative to elusive formal employment (20, 21).

Inclusive employment is a human right, so persons with mental disability have the right to employment as recommended by the United Nations Convention on the Rights of Persons with Disabilities (CRPD) (22). Equity and social justice require governments and employers to guarantee equal employment opportunities for persons with mental disability devoid of discrimination on account of illness (21). Although the World Health Organization (WHO) recommends competitive employment through Individual Placement and Support (IPS) for employment of persons with severe mental illness (23), such mechanisms are often unavailable in low-income settings. In a review by Mills, inclusion of mental health as a global priority is relevant to economic development and achievement of the Sustainable Development Goals (SDGs) (2).

However, in low- and middle-income countries where social welfare is almost non-existent, individuals with mental disabilities lack the kind of support provided by governments in high-income counties (24). The Kenya National Commission on Human Rights report on mental health highlights the complex challenges that persons with mental illness in Kenya are facing (25). The magnitude of the challenges of employment faced by persons with mental disabilities is sometimes unknown or ignored. In Kenya, the number of persons with mental disabilities continues to rise, with an increased call on policy-makers to address these issues (9). Article 27 of the CRPD bestows on state parties the responsibility to promote inclusive employment and opportunities to enable persons with disabilities to realize their right to work (22). This is the central idea of the social model of disability that considers the social environment as responsible for the impact of disability on the individual (10). The social model embodies a critical response to the medical model, which perceives the person with disabilities as someone with dysfunctions that need to be resolved by making changes to the person (e.g., medical treatment addressing an impairment). It carries the idea of a person with a disability as deviating from the norm that should be mitigated by making changes to the individual rather than to social norms (26). The social model has been strengthened by the rights-based disability movement, which advocates for the rights of people with a disability to participate in society on an equal basis. The CRPD resonates with the social and rights-based model (27). Yet there have also been calls for exploration of the role of the individual with disabilities on their return to work (28, 29).

Few studies have explored the employment experience of persons with mental disabilities in Kenya or looked into the interplay between societal and individual factors from the perspective of persons with mental disabilities. We have explored the perspectives of other stakeholders such as employers, mental health-care providers, workers in disabled persons/mental health organizations in separate articles (30, 31). This study aims to highlight not only the individual and environmental barriers to employment experienced by persons with mental disabilities in Kenya but also how they, as individuals, supported by their environment, manage against all the odds to find employment. This study is important because it offers the actual experiences of persons with mental disabilities and the factors that enabled them to overcome the many challenges on their path.

## Materials and Methods

### Study Design, Population, and Setting

We used a sequential mixed-method design (32) whereby we collected qualitative and quantitative data in the first and second phases, respectively. Study participants were recruited through two networks of persons with mental/psychosocial disabilities, namely, Users and Survivors of Psychiatry (USP) and the Africa Mental Health Foundation (AMHF). In this study, we alternated mental with psychosocial disabilities, the term preferred by mental illness rights groups. The USP is a support network of persons with psychosocial disabilities in Kenya, while AMHF is a nongovernmental organization (NGO) dedicated to research and services related to mental health in Kenya. All individuals involved in the study were clinically stable and were not actively ill at the time of the study.

### Sampling

The study participants were invited to participate in the study through the networks of USP and AMHF, and those who consented were invited for the qualitative study [in-depth interview or focus group discussions (FGDs)] and quantitative study. A total of 14 individuals participated in the in-depth interviews, while four FGDs with a total of 30 individuals were conducted. A total of 72 individuals participated in the quantitative study (20% of individuals overlap between both studies).

### Data Collection

In the qualitative study, we sought to explore the lived experiences of persons with psychosocial disability and how they were able to find employment. The interviews were conducted by IDE and a master’s student after participants were provided with the study information. Interview locations included the office of AMHF or any other location chosen by the participant. Consent was obtained from all study participants. Three of the four FGDs were conducted by trained research assistants in Swahili and translated to English, while one FGD was conducted in English by IDE. The interviews were semi-structured and explored both perceived barriers and facilitators of employment for persons with mental disabilities. The FGDs explored the same themes as the interviews but sought to generate a consensus and a validation of the themes identified in the interviews. The interviews and FGDs lasted for 30–60 min and were recorded and subsequently transcribed verbatim. Data saturation was deemed to have been achieved when no new information was obtained from the interviews and FGDs (33, 34).

The quantitative study sought to explore the factors that hinder or facilitate the employment of persons with mental disabilities in a larger group of respondents. The results from the qualitative study were used in the design of a questionnaire, which was pre-tested by the researchers and sought to validate the findings of the qualitative study. The questionnaires were administered in English or Swahili, the two official languages in Kenya. The questionnaire documented the sociodemographic characteristics of the study participants and perceived barriers to and facilitators of their opportunities of employment. The social function of the study population was measured using the Social Functioning Questionnaire (SFQ) (35). The complete details of the SFQ have previously been published (36).

### Data Analysis and Integration

The qualitative data were imported into Atlas.ti version 8 and analyzed thematically (37). The qualitative data were independently coded by IDE and EO, and the resulting coding scheme was shared with MG, JFGB-A, and BJR. All authors subsequently discussed the coding scheme after which the final themes emerged. The quantitative analysis was conducted using IBM SPSS version 23 (IBM, New York). Descriptive statistics was used to explore the sociodemographic characteristics of the study participants and their perceived barriers to and facilitators of employment. We used an iterative analytical process to ensure integration of the qualitative and quantitative data throughout the analysis. In addition, the study participants were involved in the analysis and also collaborated in the study to ensure validity and acceptance of the findings (38). The study results and their analysis were shared with some members of USP, one of whom participated in preparing the manuscript.

## Results

### Characteristics of Study Participants

In the qualitative study, FGD1 and FGD2 were mixed groups of men and women, while FGD3 and FGD4 comprised only men and only women, respectively. **Table 1** shows the demographic characteristics of participants who completed the questionnaires of the quantitative study. We recorded a response rate of 60%. The mean age of the study participants is 40.0 years, and 45.8% were 41 years and above, most were women (69.4%), and 70.8% were unmarried.

**Table 1:** Socio-demographic characteristics of the participants (*N* = 72).

Variable	Categories	Distribution *N* (%)
Age	30 years and below	10 (13.9)
	31–40 years	29 (40.3)
	41 years and above	33 (45.8)
Age in years	Mean; median; range	40.0; 38.8; 23–63
Sex	Male	22 (30.6)
	Female	50 (69.4)
Marital status	Unmarried	51 (70.8)
	Married	21 (29.2)
Number of children	None	16 (23.5)
	With children	52 (76.5)
	Missing	4
Education level	Primary and below	32 (45.1)
	Secondary and above	39 (54.9)
	Missing	1
Type of mental disability diagnosed	Schizophrenia and other psychotic disorders	12 (18.8)
	Depression	20 (31.3)
	Depression and other comorbid conditions	19 (29.7)
	Bipolar disorder	13 (20.3)
	Missing	8
Employment status	Unemployed	40 (55.6)
	Employed*	32 (44.4)
Job satisfaction (among the employed)	Satisfied	15 (46.9)
	Not satisfied	17 (53.1)
Interested to be employed	Yes	53 (76.8)
	No	16 (23.2)
	Missing	3

*Fifteen of the 32 employed participants were self-employed (36).

Regarding the mental illness types, depression was the highest self-reported diagnosis (31.3%), while schizophrenia and other psychotic disorders were the least (18.8%). The mean social functioning score of study participants was 12.8 (SD = 5.7), and a significant association was noted between impaired social functioning and unemployment [14.0 vs. 11.2 (*p* = 0.037)] (36). Over half were unemployed (55.6%); and of the 44.4% that were employed, half were self-employed. Slightly more than half of those employed were not satisfied with their job, and 73.6% of all respondents were interested in employment.

### Experiences of Mental Disability and Barriers to Employment Opportunities

In this section, we present two case studies (**Box 1**) that highlight the experience of the participants and subsequently discuss the major themes that capture their experience and barriers to employment. It is pertinent to state that the cases are mixed and do not reflect the experience of any specific individual.

Box 1 **Case Studies 1 and 2 highlighting the experience of mental illness by two participants and the barriers to employment.**

**Case Study 1**

This is the story of Nyawira, a 43-year-old woman who lives in Nairobi and was diagnosed with bipolar disorder 7 years ago. She describes how it has been very hard for her to keep a job, especially before she was diagnosed. “*I [would] just go through seasons when I could not get out of the bed. I couldn’t do anything and I didn’t know what it was. And you know, because you don’t know what is wrong, people take it that you are lazy or you are un-motivated or you are un-focused*.” This meant that often employers would not keep her. “*People would not understand, I would be late for work for say three, four, five weeks, and so they cannot handle it any more. They say ‘okay, we gave one warning letter, then the second warning letter, now we have to let you go because of lateness’ or whatever the cause.” Or she would resign herself. “I would go through say six months being fairly well and I would start wearing down, and then I would have to resign. And because I didn’t know what was wrong, I had to give some flimsy reason why I am resigning from whatever job it is*.”

After the diagnosis, there is no stability in symptoms. There are periods when she has so little energy that it is hard to even get up. “*Like today, actually the last say two weeks I have been unable to do even the most basic things like getting up, showering. I do not have enough physical energy to do a lot of things that would require me to get up, go to work, interact with people*.”

The side effects of medication also play a negative role. “*I decided I wanted to see what it would be like not to be on medication because some of those drugs were making me basically completely spaced out. I couldn’t function at all, at all, at all. [They made me] drowsy like I think I slept through one particular seven-month period of my life*.” Given the instability of the illness, the periods of depression and not being able to function properly and the limited record of job retention made it not possible to find or retain employment. She indicates: “*I don’t know any employer who will be able to work with my ups and downs*.”

**Case Study 2**

This is the story of Bahati, a 23-year-old man who was diagnosed with bipolar disorder about 7 years ago. He narrates the impact of the illness on his work. “*I started missing work, I started asking for time off. And I was the only person there at the Boutique, as well as the snack shop. I had to give out one; the snack shop I gave it out to my cousin, then I tried to work at the Boutique. But I still couldn’t*.”

One of the reasons was the symptoms associated with the illness. “*Then I [used] to move around a lot and talk a lot. And I couldn’t stay in one place and it was hard for me*.” His parents made him give up his job. “*I never disclosed anything to [the owner]” because “… he was also a family friend and my parents didn’t want word going out that I had a problem*.”

The illness also affected him at school: “*In my second year, the first semester I started going into maniac again, So when I stopped taking medication I think it affected me so I started missing school, I started missing classes, being late for classes*.” On account of it, he had to stop school: “*So it reached a point now where the principal advise[d] that I take a break from school. Which I didn’t take very lightly because I felt that they didn’t understand me and my condition. But eventually my parents talked to me and I accepted to take the break. So I took the break – it was going for six months*.” He describes his experience at home and the lack of understanding: “’*Why do you keep locking yourself in the room? Why can’t you go out like every other young guy? Why can’t you go make friends?’. So they didn’t know. Sometimes I would just go and sleep excessively. [ … ] They would take that as laziness and they would really lecture me a lot of times about that*.” He shares not feeling supported by his father “*I tell you, my dad has never [ … ] been with me to a doctor’s appointment, apart from that one time at high school. At times, I would come home, just go to my room and cry a lot and sometimes even scream. And I am hitting things. My dad would be ‘Why are you crying? You are a man you need to be strong!*’.”

Both Nyawira and Bahati share the impacts of mental illness on their functioning; they go through periods of not being able to get up in the morning and losing jobs because of it. Periods of mania, and the side effects of medication, also contributed to losing employment. Besides the direct effects of the illness, the lack of understanding and support from, and even the demeaning attitude of their family might be expected to block the route towards (self-)acceptance and developing coping strategies. Both cases also show the perpetual effects of the illness on employability—in Bahati’s case by having to take a break from his education and in Nyawira’s case by the series of short jobs and not being able to build a career because of it. She says:

*Because of that cycle of getting a job working briefly, resigning, looking for another job, one of the things that happens is that you can’t build your career. Because you’re never in a place long enough, it’s very hard to advance in terms of roles, responsibilities, how much you earn, benefits you get from your employer. [ … ] You find that at 31, 32 you have not built anything with your life and yet the people who you were in college with, have done quite well for themselves and yet, you may have had better opportunities than they did.*

Finally, not being able to disclose to the employer means no chance of the workplace accommodating the person’s abilities, and limits opportunities.

The other study participants shared similar experiences of mental illness affecting their employment opportunities. The perpetuating factors of the *mental illness* itself, *social exclusion*, and* stigma*, resulting in *work identity crisis*, as well as a *non-accommodating environment* clearly came to the fore. Further, we found *socioeconomic status* to be an underlying factor hampering the other factors that affected employability.

#### Mental Illness Factors

The experience of mental illness by most of the study participants was regarded as complex and limiting. The fluctuating nature of mental illness meant that most of them had their lives and daily activities interrupted and were taken over by both the illness and the side effects of their medications. These side effects limited both their education and socioeconomic activities and thus formed barriers to employment opportunities. Describing the effect of medications on his ability to work, one participant declared: “*You see, at times when I take my medication, I become a bit slow and sluggish and so it affects my work. So, every time at [my] work[place] and at school when I need to work overnight I don’t take my medication*” (PWMD5_Man). The side effects of the medication thus also affected adherence to treatment. Overall, participants were unhappy about the mental health services, which they felt were poor and affected their health care and well-being. They perceived their experience of mental illness to reflect the poor mental health services in their setting. Most participants despised the national referral hospital, and some declared they would rather avoid it.

*If the government cares about Mental Health, they would upgrade the [National Referral Psychiatric] Hospital. It has the poorest conditions and patients say like they are in hell. I have been there as well. You know patients sleep on the floor, they pee everywhere. There is loo everywhere, you know. Some eat their own faeces and nobody cares. You know if they refuse to take medicine they are beaten like cows*. (PWMD4_Woman)

Similarly, in the questionnaire, mental illness was the highest reported limitation to opportunities of employment (63.2%) (**Table 2**). An interviewee talks about how mental illness made her ill-suited for formal employment because she was so tired of offering excuses to her employer about her declining performance, and then she opted to quit.

**Table 2:** Barriers to and facilitators of employment opportunities.

Question	Frequency (*N*)	Percentage (%)
What are the factors that limited your opportunities of employment/job? (*N* = 40)		
Employers do not want me	13	34.2
Due to my sickness	24	63.2
I am afraid of meeting people	9	23.7
I do not have useful employment skills	12	31.6
I do not have money to set up a business	17	44.7
Others	11	28.9
*No response*	*4*	*10.0*
In what ways can employers be of help to you? (*N* = 72)		
Allowing me sick leave	44	67.7
Ensuring other workers don’t discriminate against me	43	66.2
Allowing me to have flexible work schedules	37	56.9
Others	27	41.5
*No response*	*7*	*9.7*
What factors promoted your chances of employment? (*N* = 32)		
Supportive employer	6	27.3
Supportive family and friends	12	54.5
Disability movement/Support group	6	27.3
Self-motivation to work	4	18.2
Taking my medication	4	18.2
*No response*	*10*	*31.3*
What factors can promote your chances of employment? (*N* = 72)		
Informed and supportive employer	4	6.2
Self-employment and capital for business	17	26.2
Government support and welfare services	4	6.2
Job training and skills acquisition	14	21.5
Networking and participation in support groups	10	15.4
Not interested in employment	2	3.1
Others	14	21.5
*No response*	*7*	*9.7*

*The illness was too much to cope with because there are those days when you don’t want to wake up, there are those times you are in a bad mood, I just couldn’t manage. You know I was working in the bank and the constraints are a bit high. My work was being affected and my performance kept declining all the time and I felt I was doing my best, so I just thought if my best is not good enough, I’d rather just let go.* (PWMD8_Woman)

For another participant, again obtaining a job was not the problem but keeping the job was. In the last year, she had resigned from four jobs and only later realized that her penchant for quitting jobs was part of the peculiarities of bipolar disorder.

*For me, getting jobs is quite easy but the problem is staying on the job. I really need, I want a job and I want be employed. I actually quit my job on Friday*… (PWMD9_Woman)

#### Social Exclusion and Stigma

The ignorance and myths surrounding mental disability accounted for the social exclusion and stigma experienced by most of the study participants. They not only experienced stigma but also anticipated stigma, which stopped them from completing education, seeking employment, or having intimate relationships. Their past experiences of exclusion made them feel they would always be rejected or excluded. Describing their experience, one of the women in the FGD painted a vivid picture of the ordeal of waiting in vain to be selected for work:

*… We have been segregated. We cannot be selected for the job so I don’t go. Because why should I stay one month sitting on a rock waiting for a job and I don’t get? I have wasted my time.* (FGD4_Woman)

**Table 2** shows that the fear of meeting people was among the self-reported limitations to employment opportunities or having meaningful relationships. The social exclusion also meant that they had reduced social networks and also treated unfairly by their family, community, and co-workers.

*The treatment I have experienced from community is hate and rejection, stagnating and [hence] no progress academically, professionally, socially. It was like I was somewhere in a cocoon or in an enclosure somewhere.* (FGD2_Mixed)

This experience also occurs in religious organizations where participants expected succor but lost positions on account of mental illness. According to one participant, her position in the church was terminated after she disclosed her illness.

*In fact, the most place that I experience stigma is at church. So I think, I think the church has failed in terms of mental health. So there was a time I was chosen as a leader and when my name was presented to the leaders, they said this lady is of unsound mind so I felt so bad. But I didn’t answer them back although I sent somebody to go and tell them. So I stopped doing church activities. I was teaching the church Sunday school. I stopped teaching in the church school. I just go now for the meetings and I just go home. But I don’t take part in anything.* (PWMD4_Woman)

The social exclusion and stigma led to the decision not to disclose even though they know that disclosure would grant them the support they needed. Of the 14 persons involved in the interviews, only six had disclosed their status to their present or past employers. The consensus opinion in the FGD was that disclosure during an interview was bound to affect work opportunities because the employer’s response may depend on his or her attitude to mental illness. For most of the study participants, self-disclosure of mental illness was associated with negative reactions from society and further isolation. Hence, most preferred not to disclose or share their problem.

*…there is a problem in opening up and saying I suffer from mental illness because most people think, it’s called madness. So you are stigmatized at work, you do anything that is a normal mistake for anyone, but everyone goes like “no leave that one she’s got this problem”.* (PWMD1_Woman)

#### Work Identity Crisis

Persons with mental illness are sometimes perceived as not fit or able to work. This myth is often shared by persons with mental illness, leading to self-doubt in their perception of their ability to work. The participants identified a work identity crisis as a limitation to opportunities for employment. This was perceived as related to the self-doubt and reduced self-esteem that they experienced on account of mental illness. While some of the participants identified the debilitating nature of the illness as the problem, others suggested that it was a result of the social exclusion they experienced that forced them to believe that they are unable to work. A participant in the FGD with USP described his experience: “*There were jobs I refused to go because I was afraid. I recall they could invite me and I could not even engage myself. Yeah others I could leave halfway and there are others I could do very incompetently that they would not want to see me back* …” (FGD2_Mixed).

Similarly, a participant in interview narrated her inner wish that she would not be employed and her belief she is not capable of work.

*I was going for an interview, but deep down in my heart I was like, I hope I am not chosen because if I am chosen and I go for an interview and I don’t do well my world would be shattered.* (PWMD1_Woman)

#### Non-Accommodative Environment

Also related to social exclusion and stigma is the non-accommodative nature of the socio-political environment. Although persons with other disabilities were sometimes recognized and assisted in society, this was different for persons with mental disabilities. The misconception of and biases regarding mental illness thus constitute a barrier to education, health, and employment. The majority of the participants had their education interrupted by mental illness and were not extended the accommodation that they deserve in the same way as other persons with disabilities.

*There is discrimination, because … just the way they make sure that there are ramps for people with wheelchairs to walk on, they should also provide ways in which somebody with a mental illness is able to cope at their level. And then also with the medication…* (PWMD8_Woman)

This same attitude was found in the health sector where they faced challenges from insurance companies that refused to allow them to take out a policy and health-care providers that treated them unfairly. Regarding the insurance companies, a participant in the FGD declared: “*So, I think there is a problem at the policy level and the treatment level and also the insurance companies are also very discriminatory but accommodating for other diseases* …” (FGD2_Mixed). Narrating her ordeal in a public hospital, one participant stated:

*One time, I was so depressed, I was like so suicidal and I just wanted like to get back on my medicines. So I went there at around 4.30 and they told me that the doctor cannot see anyone else because she is supposed to – uhm –normally it is supposed to open from 8 to 5, so, this is at 4:30 and they are telling me that I cannot see the doctor because I came in late. That was like a huge blow.* (PWMD9_Woman)

In the workplace, the study participants recounted stories of termination on the disclosure of their illness. This non-accommodative work environment was perceived to be worse in private organizations than in public or government-owned organizations where the bureaucracy sometimes protected them from being sacked.

*In my experience, it is better to work in public rather than private. In the private sector, if you make a mistake, they sack you immediately; there is no process but sacking somebody in the public sector is quite a process.* (PWMD4_Woman)

The negative attitude of employers was reported by 31.6% of study participants as the major limitation to their being employed (**Table 2**).

#### Socioeconomic Status

The study participants identified socioeconomic status as a major determinant of their experience of mental illness. This was because it determined if they were able to buy their medications or access hospital care, buy food, complete their education amidst the interruptions, or harness self-employment as an alternative to employment. These feelings of helplessness were described by a participant in the FGD with members of USP.

*Actually now here it depends on the social strata or the economic status of an individual. There are those people who can afford to seek the private health services. But there are very many people who don’t have the choice, of where to go so they are just ushered in to the [National Referral Psychiatric] Hospital*. (FGD2_Mixed)

The financial challenges faced by participants of low socioeconomic status were so enormous that they were unable to buy medications even when they wanted to. One participant described the choices some of her friends had to make to buy food rather than spend the money on medications, because the hunger for food was greater than that for medication.

*…I have worked with people from lower socioeconomic status and I have seen when they have to decide between medication and food … which is it either or you know yeah. So that sort of choice I never had to make … knowing that I will wake up and there would be sort of food waiting and being able to go to the hospital and keep getting more medications. So, I would say that also helped in a way just … that sort of social economic status. I would say helped in a way.* (PWMD13_Woman)

This dire financial challenge was also noted in the responses of participants who completed the questionnaire. The lack of money to set up a business was the second-highest reported limitation to opportunities of employment (44.7%) (**Table 2**). The lack of access to capital was summed up by a participant in the FGD: “*most of the people here have skills. They are very skilled; but getting capital is the problem*” (FGD1_Mixed).

Factors hampering employment are closely intertwined, and when analyzed through the individual versus environmental lens, we see perpetuating effects of both. Looking at the inseparability of individual and environmental factors, it would be hard to argue that responsibility for facilitating employment of people with a mental disability lies solely with society, or solely with the individual. While stigma plays a part in exclusion from education, or health care (which in turn leads to sustained symptoms), it also leads to anticipated stigma and self-stigma, which prevents people from finishing education or looking for employment. Hence, there is no single, unequivocal starting point for improving the employability and employment of people with a mental illness. Just like the different pathways through the individual–environmental nexus that lead to low employment of people with a mental disability, different pathways may be identified that may facilitate employment.

### Factors That Facilitated or May Facilitate Employment Opportunities

Once more, using the case studies discussed previously, we highlight the factors that facilitated the employment experience of the individuals (**Box 2**). Subsequently, we discuss the major themes that facilitated or may facilitate employment based on the experience of the study participants.

Box 2 **Case Studies 1 and 2 highlighting factors that facilitated employment.**

**Case Study 1**

Nyawira solved her employment challenges by embracing self-employment. She now runs a small business selling beauty products and is planning on starting a school bus company. She stated that: “*Once I understood what was wrong with me and what needed to work, I mean how I needed to figure, I mean what I needed to figure out in order to be financially stable, I started a business. So I run a small business I sell beauty products and the reason why it works for me is because I do deliveries.*” What helped her in this transition was: “*I understand a lot of financial instruments, so one of the things that once I accepted my diagnosis, I figured out OK, so clearly the workplace will never really work for me … I actually have to be disciplined enough to put aside money, the second thing was access to credit which I think was one of the biggest hurdles for me and I knew I was not creditworthy with the bank because I don’t have a job and my business is not big enough for them. So I started, I looked for a SACCO I could join, and I found one and I joined and after I think about a year and two months, I was able to take my first loan and I bought a car for the business*.”

In addition to her knowledge of finance, she avers that self-awareness and motivation also helped her in her journey to self-employment: “*once you are brutally honest with yourself then what happens is you are able to do your best, you are able to push yourself as far as you can*.” Also, the support from her family helped her cope with her illness and engage in self-employment: “*I have a very supportive husband and he sometimes does my deliveries for me*.”

Family and social support was also related to education and social status: “*Because for me the fact that the people around me are probably on the same socioeconomic level, means that they, they have a much better understanding of the mental illness and so they are able even if they don’t understand it completely, they are able to give me more, a leeway to work around my limitations*.”

**Case Study 2**

Bahati found employment with an accommodating employer: “*Then I told them that I am actually bipolar … that’s when he also told me that he also had a reading disorder. So he explained to me how for him it was for him, how he worked with it and the challenges he faced in school and how he even came to start working*.” He also describes the provisions his boss made for him: “*They are paying for my whole entire fees. They are paying for my projects and also they gave me a job. So they told me after, when I finish school, I have a job there. So it was, for me it was positive because I, I felt like, I felt somehow inadequate while working there cause I didn’t feel I was good enough*.”

Joining a support group changed things for him: “*So being there really helped me. You see with my friends I can’t tell them how I feel, I can’t tell them how I am at certain period. Because it’s hard for them to tolerate what I am actually going through, because none of them have what I have and they can only do so little. But with someone who actually has the same if not a similar condition as you are it’s a very different way. Because if you talk with them they actually understand*.”

He reflects on the usefulness of self-awareness and motivation: “*Then I realized that very few people would care whether I am sick or not. You have to deliver. If I have employed you, you have to deliver whether you are sick or not. I know you are sick but you cannot keep on asking for time off and yet you are getting paid. It’s your job, you either work hard on it or you are going to lose it. And getting another one is a big problem, it’s a big challenge. Especially someone fresh from School, it’s a big challenge. So for me whether I was sick or not I used to go to work and I used to work. And I used to make sure that I deliver. So I knew I had something slowing me down, I knew I had a challenge with my health. But regardless of that I made sure that I do my best, I do my best*.”

In spite of the challenges faced by Nyawira and Bahati, they managed to find different pathways to fulfill their needs for employment. For Nyawira, her knowledge of finance, education, and family support made it possible to engage in self-employment. For Bahati, finding an employer who was willing to offer him reasonable accommodation saved the day. It is pertinent to note that his disclosure to his boss was not spontaneous. He stated: “*now there came this day I broke down at work, so that’s when I had to tell my boss, actually I had to apologize for not telling them, because after all they were like, I was in their hands when I am working there. If anything happens to me they would be held accountable*.” His experience also highlights the unpredictability of an employer’s reaction to disclosure and that it is not always negative. It is pertinent to note that both Bahati and Nyawira share the importance of self-awareness; it was after understanding their illness and accepting that it was there to stay that they learned to deal with the symptoms and found the strength to push themselves further.

Other study participants also shared their experiences of factors that facilitated employment or improved employment opportunities. These factors include *self-awareness and acceptance*, *self-employment*, *provision of reasonable accommodation*, *improved health services*, *addressing discriminatory laws and practices*, and *social development programs and support*. We observed that self-awareness and acceptance of illness were very relevant to recovery, coping, and the decision to work.

#### Self-Awareness and Acceptance

Study participants spoke of self-awareness and acceptance of the illness as a major turning point in their lives and also in the bid to secure employment. They suggested that personal understanding of their illness motivated them to overcome the burden of illness opt for employment. One participant recounted how self-acceptance helped him to move on: “*I came to the understanding that this is how I am and probably I might be like this for the rest of my life so I rather to come to terms with it or and deal with it or continue suffering*” (PWMD5_Man).

This view was also echoed by another participant who stated:

*…the first things is that you accept yourself; in fact it is the most important thing because when you accept that you have a challenge, you have a mental illness, you will know that you may never leave medications. Sometimes you have to make painful decisions which will cost you dearly. The world would not understand you, the people around you do not have the knowledge that you have about you.* (PWMD7_Man)

Although qualitative data suggest that self-awareness and acceptance are key to self-motivation, only 18.2% of the participants in the survey who were employed identified self-motivation as one of the factors that promoted either employment or self-employment (**Table 2**).

#### Self-Employment

Like Nyawira, many study participants considered self-employment as flexible and viable to escape the challenges of the formal work environment and fluctuating pattern of mental illness. The relevance of self-employment in the employability of persons with mental disability was reported by most study participants. Self-employment was conceived as an alternative to formal employment, which they were unable to secure or is difficult for them to endure owing to the challenges specific to their illness. Participants recounted how they gave up formal employment for self-employment because it offered them more peace of mind.

*…so I left my job and decided not to seek employment. Even when I have a job, getting to work is not all that easy. So, I chose to be in self-employment so that I can sleep all I like and I don’t have that pressure of time.* (PWMD11_Woman)

Of the 14 interviewees, five were self-employed and four spoke of their intention to give up their formal employment for self-employment.

Self-employment and capital to set up business were the highest reported facilitator of chances of employment among study participants who completed the questionnaire (**Table 2**).

On self-employment, one participant declared: “*Then about the self-employment I think that’s perfect work for people like us. It’s more flexible*” (FGD1_Mixed).

#### Provision of Reasonable Accommodation

Self-employment is not an option for everybody, and even for persons without disabilities, it may be challenging. As employment is a human right, both the government and employers have a duty to provide reasonable accommodation to facilitate employment for persons with disabilities. The provision of reasonable accommodation was, indeed, identified as a facilitator of employment and includes education, employment, and health-care services. Given the fluctuating nature of mental illness, participants suggested that policies that ensure reasonable accommodation in education would assist them to acquire an education in spite of their illness. One participant recounted the accommodation provided by his school to enable him to continue his education despite the challenges of his illness.

*…and it’s good that the school has been very cooperative, I mean they understand my situation. So, they gave me the break for two weeks. I just took a rest was able to complete my projects during that time….* (PWMD5_Man)

Among those who were employed, reasonable accommodation in the workplace in the form of a supportive employer amounted to what ensured employment. According to one participant:

*…working in a big and supportive company that provided medical cover helped. There was a time when admission was the order of the day so if that was not provided then it would have been difficult for me to access care…* (PWMD8_Woman)

Asked how employers may be of help to them, allowance of sick leave (67.7%) and ensuring that other workers do not discriminate against them (66.2%) were the needs most reported by the participants who completed the questionnaire.

In order to improve employability, there needs to be reasonable accommodation (e.g., allowing for sick leave in education and employment). Similarly, the participants suggested that owing to the overwhelming nature of their illness and nature of health-care services, policy-level interventions that would ensure the right to health would improve both access to and uptake of health care, which would strengthen their workability.

#### Improved Health-Care Services

The pivotal nature of the health system in facilitating employment was reported by most of the study participants. They suggested that having affordable and appropriate medications and mental health care could make a difference. Among those who were working, compliance with medications and their availability were suggested as very important to their workability. When asked about the most important factor that helped workability, one participant stated: “*I think the first is just getting treatment, …not just treatment but getting the treatment that works for you … I think treatment should be made much, much cheaper*” (PWMD10_Woman).

They also suggested that the availability of the effective medications in the public hospitals would make it possible for them to obtain them. One participant narrated her experience with cheap medications:

*… So, I went back to the cheap drug, that one only costed me two shillings … but you see it’s not having very nice side-effects. But when I relapsed in 2014, actually the reason why I relapsed is because I was so fed up with that medication, the cheap drug. You know it was hurting me mentally and physically. There were so many things I couldn’t do. You know I am a writer, I couldn’t write, I couldn’t write.* (PWMD11_Woman)

Among those who were employed, taking their medications (18.2%) was identified as one of the enabling factors for employment (**Table 2**).

#### Addressing Discriminatory Laws and Practices

The participants suggested that addressing the discriminatory laws and practices that are rife in the country would ensure inclusive employment practices. Participants stated that as long as the laws were discriminatory and used stigmatizing language such as unsound mind, it would be difficult for employers to consider them for employment. This was aptly captured by a participant who retorted: “*Who is going to employ you if they believe you have mental illness because you use drugs or are crazy*?” (PWMD12_Woman). This statement also captured one of the challenging misconceptions that every mental illness was related to drug use. Participants suggested that the non-implementation of policies on inclusive employment was a barrier to their employment. One such discriminatory practice was the red tape surrounding the acquisition of a disability certificate, which is so much more difficult for persons with mental illness than for persons with other disabilities.

*…getting the disability card has some benefits for persons with disability. But completing the medical assessment takes up to six months for person with mental disability. Reducing this time would encourage people to go for the card and help them in the search for job…* (PWMD14_Woman)

Participants recommended better mental health-care services in public hospitals and identified the role of the government in ensuring equitable care.

*The government can also put some regulations in public hospitals so that there are services for the mentally disabled so that they can be treated and can get jobs without being looked at as less able or incompetent simply because they have not gotten the services from the hospitals.* (FGD3_Man)

The overall improvement in attitudes to mental illness through information was deemed as relevant to improved employment for persons with mental disabilities. This was noted as critical to a change in discriminatory policies and practices. One participant stated: “*The government is the one that needs to set the ball rolling in terms implementation … we have the policy but we need the implementation and follow-up. People need to be educated and informed about mental illness* …” (PWMD13_Woman).

#### Social Development Program and Support

The participants identified social development and support as useful for improved employment opportunities for persons with mental disabilities. Most decried the lack of government social welfare provisions, and how these would address the inequity they face because of mental disability. Government support and welfare services were among the factors participants said might improve their chances of employment.

*So I think the government is the one who could help….Because if they put legislation can help you when you are sick or just create opportunities for employment and they can also ensure people are accommodated and get equal opportunity in employment.* (PWMD11_Woman)

Participants also identified the provision of welfare services by the government helpful for employment or self-employment. Similarly, participants in the questionnaire survey suggested that provision of job training and skills acquisition (21.5%) would facilitate employment opportunities (**Table 2**).

Social support from families, friends, and mental health support groups was described as invaluable to employment. Among those who were employed, supportive family and friends were the highest reported enabler of employment (54.5%) (**Table 2**). According to one participant, without the support of his family, he would not have completed education or be employed: “*My family helped me … OK even my mum always asks me if I have taken my medication. They are supportive, you know family is your family. Your brother will be your permanent friend. Your sister will also be your sister*.” (PWMD6_Man).

Also, participants identified networking with support groups as one of the facilitators of employment and coping with mental illness. One participant stated: “*USP Kenya has helped me, because I got a crowd where I know it is not only me. Because when you are alone you only think it is only you. You know I thought, I came to know it is not only me, it is a disease that many people have. And people do work, and people are educated*.” (PWMD4_Woman).

To conclude, factors relevant for improving the situation are not solely dependent on the individual or the environment but are interrelated. In spite of self-awareness and a personal decision to work, it would still be difficult to function in settings where an individual is denied basic health care and reasonable accommodation in the workplace or where cultural beliefs and attitudes to mental illness deprive individuals of their fundamental human rights to social benefits and a social network. Conversely, if environmental factors are in place and individuals do not wish to work because of anticipated discrimination or self-doubt, employment rates would remain low.

## Discussion

In this study, we set out to explore the lived experiences of persons with mental disabilities such as Nyawira and Bahati and how they have managed against all the odds to secure and stay in employment. In order to achieve our study objectives, we identified several complex and limiting experiences that were conceived as barriers both to daily activities and to employment. In addition, the study participants who were employed identified the factors that facilitated their opportunities of employment; all study participants suggested perceived facilitators of employment opportunities. It is pertinent to state that the complex interaction of individual and environmental factors was conceptualized as both a barrier to and a facilitator of employment.

Our study showed that mental illness was the highest self-reported barrier to employment opportunities. This perception was related to the debilitating nature of the illness experienced, the side effects of medications, its propensity to deprive affected individuals of education needed for employment, and the reduction of their social network. Our findings in Kenya add to the established relationship between psychiatric illness and unemployment and the capacity of the illness to be a direct limitation to work (19, 36, 39, 40). It is pertinent to state that our observation from the field and the stories from the qualitative study also showed that the effect of mental illness on the individual may be independent of the severity of the illness. We had respondents with anxiety disorder who cannot hold a job because the sound of the office phone makes them jumpy. We also met persons with schizophrenia who were successfully employed and even owned houses of their own.

We noted heightened reports of social exclusion and stigma experienced in education, thus denying the persons with mental illness of the education that they need for employment. The stigma also affected them in their experience of health care, since maltreatment was prevalent in the few mental health hospitals in the country. Lastly, social exclusion also occurred among employers and co-workers, making them give up work in formal settings on account of anticipated stigma. These findings are corroborated by studies in high-income countries, but what makes our study different is the absence of mechanisms in our setting to ensure reasonable accommodation for persons with mental disabilities (30, 41–43). The absence of these mechanisms also highlights the heightened effect of these experiences on work opportunities. Despite legislation, few civil mechanisms are in place to ensure that affected individuals receive redress unless they approach the courts (5), which may be even harder for individuals without the means to buy food or medication. These observations underline the impact of socioeconomic status on the overall experience of mental disability, which the study participants also identified as a limitation to employment opportunities. Studies agree on the impact of poverty on disability and its role in worsening the experience of mental illness (1, 6, 44, 45).

Our study is replete with stories that point to a work identity crisis and the feeling of reduced self-worth. Studies have linked this to anticipated discrimination and hence the recommendation by Thornicroft and colleagues for addressing self-esteem in stigma-reduction interventions (41). However, it may be worthwhile to also consider it as a feature of the psychiatric illness or an individual’s personal decision not to work. Self-motivation was reported as one of the facilitators of employment by the study participants who are employed. Some of our study participants indicated they were not interested in working, and this line of thought is supported by a recent study that suggests that not all persons with disabilities desire to work and should not be pressured to do so (46). Although this seems to support the need for classification of personal factors (as possible barriers and enablers) in the International Classification of Functioning, Disability and Health (ICF) as suggested by Escorpizo and colleagues (47), it may also lead to governments shirking of their responsibility. Nevertheless, Mulvany (2000) recommends acknowledging that individuals differ and persons with mental disabilities may also differ in their desire for work (10). What we may be unable to confirm is whether the (reduced) desire for work is due to the illness or part of its symptomatology. Our study does suggest that social and health systems that support the individual on their journey to acceptance are greatly needed.

Our study suggests that persons with mental disabilities can work if they receive support at home, school, hospitals, the workplace, and indeed in all spheres of life. Having supportive family and friends were the highest reported facilitator of employment among those who were employed. This finding, which is supported by studies on the importance of social networks and capital for persons with mental illness, portends good news (48–50). It shows that harnessing support in our immediate families and community may improve the employment experience of persons with mental disabilities. It is related to the provision of reasonable accommodation in the workplace and health-care sectors, which our participants suggested as facilitators of work opportunities. Evidence of the importance of reasonable accommodation in both employment and a return to work schemes has been documented (17, 51, 52). The pivotal nature of improved health services through provision of universal health coverage and non-discriminatory insurance schemes cannot be over-emphasized. The provision of friendly and non-discriminatory health services, and functional procurement of essential medicines would go a long way in reducing the side effects arising from using cheap and out-of-date antipsychotics with a broad spectrum of side effects (53). This is in line with the recommendations of the Convention on the Rights of Persons with Disabilities (CRPD) and the SDGs to ensure equitable health care for person with disabilities (2, 22).

The role of government in all these areas is highlighted in the suggestions made by study participants on the need to address discriminatory laws and practices and providing development programs. Affirmative action is essential because, as the participants noted, there are laws in Kenya, but there is an absence of political will to implement them, including social welfare for persons with disabilities (9). This policy–practice gap affects work opportunities for persons with mental disability. The existence of discrimination in identifying mental illness as a disability and easing the process for acquiring a disability card in Kenya would ensure that affected individuals receive the reasonable accommodation they deserve. A study in South Africa has also documented the challenges in accessing the disability certificate for persons with psychiatric disabilities (54). Participants in the study suggest the need for social development programs to enable them to acquire skills and engage in self-employment. The establishment of social development programs would also provide individuals who want to opt for self-employment to be helped in their efforts to set up economic activities. The importance of self-employment for persons with disabilities has been previously documented (18, 21)—hence the promotion of village savings and loans as a means of capital generation and economic empowerment for persons with disabilities by the Christian Blind Mission (55). Ostrow and colleagues suggest that self-employment for persons with psychiatric disabilities has advantages such as self-care, choice of career, and additional earnings; but they also noted that it is fraught with challenges and sometimes difficult to sustain especially where stigma and lack of social support exist (18). There is need for the government to support community-based rehabilitation (CBR) programs for persons with mental disabilities in Kenya to engage in self-employment and entrepreneurship in line with Article 27 of the CRPD (22).

Our study is one of the first in Kenya that has set out to explore the employment challenges of persons with mental disabilities. The strength of our study lies in using the case-study approach and the involvement of the study participants in the study and analysis. Thus, it ensured that the voices and messages of the participants took precedence over the yearnings of researchers. Also, the exploration of our study question through qualitative and quantitative means ensured a validation of our study findings. However, these findings are not generalizable on account of our limited sample size. It is also pertinent to state that participants’ stories may have been affected by recall bias or social desirability. In addition, our findings reflect the perspectives of the study participants and may have missed the views of non-participants.

## Conclusion

Our study has highlighted that persons with mental disabilities in Kenya can work. It has laid to rest the belief of employers and certain social segments that they cannot work. We have also shed light on the various challenges (personal and environmental) affected persons encounter in their quest to enjoy their fundamental human right to employment. The problems are many, but they are not impossible to overcome. Our study holds promises of improvement if they receive support from their social networks. The fulfillment of government obligations is pivotal to the enjoyment of reasonable education, health care, and employment for persons with mental disabilities in Kenya.

## Data Availability

The datasets generated for this study are available on request to the corresponding author.

## Ethics Statement

The approval for the study design was granted by Amsterdam Public Health (WC2017-011), and ethical approval was obtained from Maseno University Ethics Review Committee (MSU/DRPI/MUERC/00391/17).

## Author Contributions

IDE, BJR, and JFGB-A were involved in the research design. IDE collected the data and analyzed it with MG, EO, JFGB-A, and BJR. IDE wrote the initial draft, which was revised by MG, EO, JFGB-A, and BJR. All authors approved the final version of the manuscript for submission.

## Funding

This work was supported by funding received by the first author from the Erasmus Mundus Joint Doctorate (EMJD) Fellowship-TransGlobal Health Consortium FPA 2013-0039 (SGA2016-1346).

## Conflict of Interest Statement

The authors declare that the research was conducted in the absence of any commercial or financial relationships that could be construed as a potential conflict of interest.
